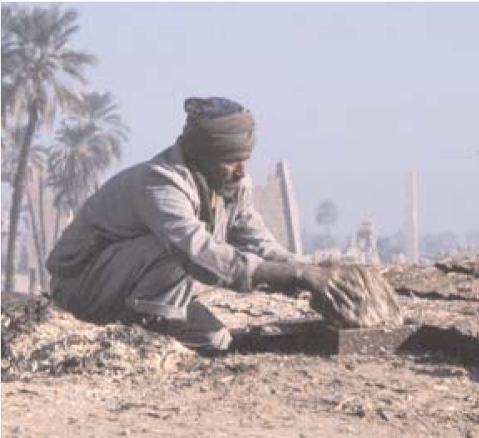# The Beat

**Published:** 2007-12

**Authors:** Erin E. Dooley

## O Happy Day for Malaria Treatment

A new understanding of malaria pathogenesis could guide the development of new antimalarial drugs. A study published in the 30 October 2007 issue of *Proceedings of the National Academy of Sciences* showed that people with blood type O were 66% less likely to experience severe, life-threatening cases of malaria. In type O patients, rosettes—obstructive clumps of infected red blood cells bound to uninfected cells—occurred less frequently; rosettes that did occur were not as well formed as those in patients with other blood types. The authors believe a future vaccine could promote antibodies that inhibit rosetting and thus prevent some cases of severe malaria.

**Figure:**
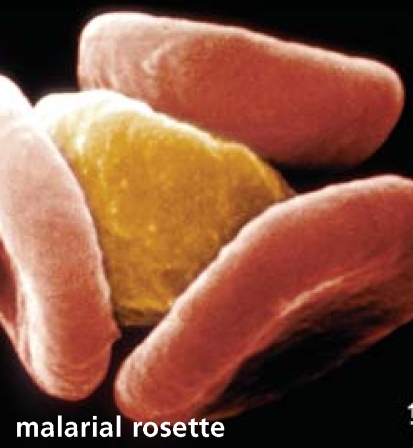


## Breastfeeding Trumps DDT

Given that a number of organochlorine pesticides such as DDT can be passed from mothers to infants through breast milk, breastfeeding has been questioned as the best way to feed babies of exposed mothers. Pre- and postnatal exposures to these chemicals havebeen linked to disruptions in neurologic development that can affect mental and psychomotor functioning. However, in a report in the October 2007 issue of the *American Journal of Epidemiology*, Spanish researchers showed that, regardless of DDT exposure prenatally and via breast milk, children who were breastfed for more than 20 weeks performed better on tests of verbal skills and memory at age 4 years than peers breastfed for shorter periods.

## Artisanal Diamond Mining Gets a Hand

Although artisanal diamond mining doesn’t use toxic chemicals such as mercury in the way artisanal gold mining does, the process does contribute to deforestation and remove tillable land from cultivation, and is associated with higher rates of diseases such as malaria and schistosomiasis. The Diamond Development Initiative, founded in 2005 to improve working and economic conditions for the more than 1 million artisanal diamond miners in Africa, announced in October 2007 that the Swedish Ministry of Foreign Affairs and The Tiffany & Company Foundation will provide grants totaling more than $750,000 in support of initiative programs. The monies will provide the initiative with core operating support for its work as well as funds for a standards and guidelines project on artisanal diamond mining.

**Figure:**
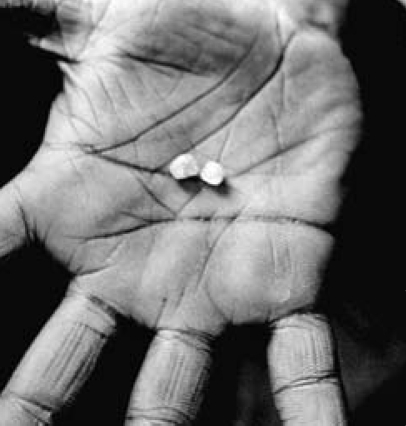


## Folic Acid Lowers Blood Arsenic

Arsenic-contaminated drinking water occurs in at least 70 countries, and chronic arsenic exposure, which currently affects 100 million people worldwide, is linked with adverse health effects including certain cancers and cardiovascular disease. A study conducted in Bangladesh that appears in the October 2007 *American Journal of Clinical Nutrition* finds that folic acid supplementation in populations deficient in this B vitamin reduces total blood arsenic levels by 14%. The folic acid helps the body convert a toxic metabolite of arsenic, methylarsonic acid, to a form that is more easily excreted. The authors note that folic acid supplementation may also reduce stores of arsenic in the body that remain after exposure ends.

**Figure:**
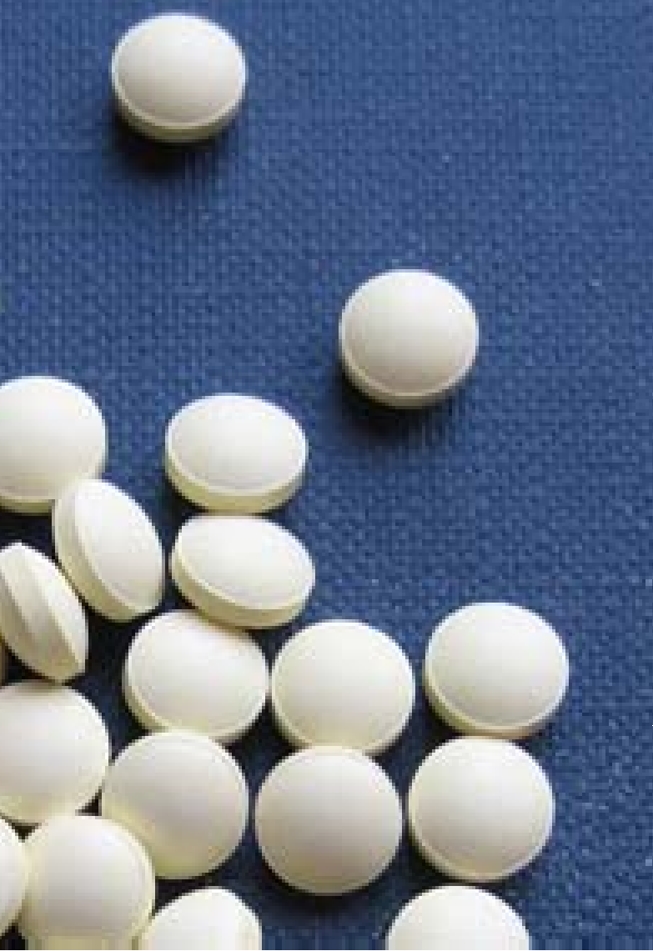


## Child Asthma Hospitalizations Plunge in NYC

A September 2007 report from the Office of the New York City Comptroller outlines a number of health disparities among the city’s income groups. Despite several negative findings, one statistic is positive: hospitalization due to asthma-related causes among children from the poorest third of the city dropped by around 50% from 1995 to 2005. Some experts attribute this decline in part to city policies that took aging diesel buses off the streets and replaced them with cleaner diesel, natural gas, and hybrid electric models. Another factor in this trend may be the city health department’s Asthma Partnership, a broad-based network of more than 300 groups and individuals that sponsors workshops on topics including asthma management and indoor air quality.

## Making Over Egypt’s Mudbrick Industry

Cairo, Egypt, has some of the world’s worst air pollution, partly due to local factories’ use of mazot, a petroleum by-product that when burned releases substantial amounts of greenhouse gases and nearly 60 other pollutants. A Canadian agency has teamed with a group of Cairo’s mudbrick factories—some of the biggest mazot consumers—to convert them to natural gas. This change is not only reducing pollution but doing so at a profit, plus the gas-fired bricks are of much higher quality than their mazot-fired counterparts. Each of the 50 factories that have already been converted has seen a 37% reduction in yearly greenhouse gas emissions; the conversion of this group of factories provides the air improvement equivalent of taking 300,000 cars off of Cairo’s roads annually. Another 311 factories are now in line for conversion.

**Figure:**